# Dopamine Modulation of Avoidance Behavior in *Caenorhabditis elegans* Requires the NMDA Receptor NMR-1

**DOI:** 10.1371/journal.pone.0102958

**Published:** 2014-08-04

**Authors:** Melvin Baidya, Marx Genovez, Marissa Torres, Michael Y. Chao

**Affiliations:** Department of Biology, California State University San Bernardino, San Bernardino, CA, United States of America; Institute for Interdisciplinary Neuroscience, France

## Abstract

The nematode *C. elegans* utilizes a relatively simple neural circuit to mediate avoidance responses to noxious stimuli such as the volatile odorant octanol. This avoidance behavior is modulated by dopamine. *cat-2* mutant animals that are deficient in dopamine biosynthesis have an increased response latency to octanol compared to wild type animals, and this defect can be fully restored with the application of exogenous dopamine. Because this avoidance behavior is mediated by glutamatergic signaling between sensory neurons and premotor interneurons, we investigated the genetic interactions between dopaminergic signaling and ionotropic glutamate receptors. *cat-2* mutant animals lacking either the GLR-1 or GLR-2 AMPA/kainate receptors displayed an increased response latency to octanol, which could be restored via exogenous dopamine. However, whereas *cat-2* mutant animals lacking the NMR-1 NMDA receptor had increased response latency to octanol they were insensitive to exogenous dopamine. Mutants that lacked both AMPA/kainate and NMDA receptors were also insensitive to exogenous dopamine. Our results indicate that dopamine modulation of octanol avoidance requires NMR-1, consistent with NMR-1 as a potential downstream signaling target for dopamine.

## Introduction

A basic function of the nervous system is to confer the ability to detect external stimuli and to generate appropriate behavioral and physiological responses. These responses can be modulated when parallel streams of information are provided to the nervous system and neural activity is appropriately altered. A classic example of this is the effect of addictive narcotics such as cocaine via a dopaminergic pathway on glutamatergic synapses in the ventral tegmental area [1]. Another example is the modulation of silent synapses in nociceptive interneurons in the spinal cord via descending serotonergic output from the rostroventromedial medulla [2].

In the nematode *Caenorhabditis elegans*, the presence of food has a strong modulatory influence on many behaviors. For instance, the physical sensation of bacterial food stimulates *C. elegans* mechanosensory neurons to release dopamine [3]–[5], and dopamine affects many behaviors include locomotion rate [5], mating [6], foraging [7], and response to soluble repellants (e.g., Cu^2+^) [8]. In this study, we report that *C. elegans* avoidance response to the noxious stimulant 1-octanol is also modulated by dopamine.

The *C. elegans* avoidance response has been well studied. Under laboratory conditions *C. elegans* are typically raised on agar plates and spend most of their time crawling forward. When they detect various types of noxious stimuli, they respond by rapidly initiating backward locomotion. These stimuli include toxic volatile odorants such as octanol [9], soluble bitter tastants such as SDS or quinine [10], [11], heavy metals [12], [13], osmotic pressure [14]–[16], mechanical force [17], [18], and noxious levels of heat [19]. Many of these responses are mediated (at least in part) by the pair of ciliated sensory neurons named ASH that have sensory openings at the anterior amphid pore at the nose of the animal and are thought to be analogous to polymodal nociceptive neurons in vertebrates; other sensory neurons (e.g., ADL, AWB, ASK) are thought to play minor and auxiliary roles in avoidance responses. The avoidance response to 30% octanol via ASH neurons is enhanced by serotonin; furthermore, altering serotonin levels results in ADL and AWB neurons being recruited to detect 100% octanol redundantly with ASH neurons [20]. In addition to serotonin, this modulation requires a complex network of monoamines and neuropeptides, including tyramine, octopamine, and NLP-3, that function across several neurons [21]–[23].

The ASH neurons make glutamatergic and neuropeptidergic synapses with five pairs of premotor interneurons (designated AVA, AVB, AVD, AVE, and PVC) [18] that express several types of ionotropic glutamate receptors, including the AMPA/kainate subunits GLR-1 and GLR-2, and the NMDA subunits NMR-1 [24] and NMR-2 [25], which are thought to heterodimerize to form the functional receptor protein. Mutations in *glr-1* result in animals that are defective for response to nose touch [26],[27], a stimulus that is in part detected by ASH. Similarly, mutations in *eat-4*, which codes for a vesicular glutamate transporter expressed in ASH and other glutamatergic neurons [14], result in a loss of response to sensory stimuli. Thus, the primary sensory transduction pathway is, at least in part, mediated by glutamatergic neurotransmission. ASH neurons have dense core vesicles at their synapses and express many different neuropeptide genes [28]. Neuropeptides encoded by *nlp-3* likely play a role in the primary pathway for octanol response [21].

In mammals, interactions between dopamine signaling and NMDA signaling have been well characterized (reviewed in [29]). For instance, D1 type dopamine receptors can signal through classic second messenger pathways, including cAMP/PKA [30] and phospholipase C/Ca^2+^/PKC [31], [32], both of which lead to increases in NMDA responses. By contrast, D2-type dopamine receptors cause decreases in NMDA responses [33], [34]. Dopamine receptors can also physically interact with NMDA receptors to regulate their activity. For instance, in rat hippocampal neurons the C-terminal domain of the D1 dopamine receptor directly interacts with NMDA receptor subunits NR1-1a and NR2A. The first interaction directly inhibits NMDA currents, whereas the second interaction attenuates NMDA-mediated excitotoxicity via PI-3 kinase [35]. In the striatum, D1 receptors co-immunoprecipitate with NR1 NMDA subunits, and D1/NR1 complexes may play a role in receptor trafficking [36].

Herein, we characterize a novel genetic interaction between NMDA and dopamine signaling in *C. elegans*. We show that the *C. elegans* avoidance response to 100% octanol is modulated by dopamine. Wild type animals respond robustly to octanol, whereas *cat-2* mutant animals, which are deficient for dopamine biosynthesis, respond with increased response latency. This defect is fully restored with exogenous dopamine. Whereas the AMPA/kainate receptor subunits GLR-1 and GLR-2 are dispensable for dopamine sensitivity, the NMDA receptor subunit NMR-1 is absolutely required. The dopamine receptors DOP-1, DOP-2, and DOP-3 are redundantly required for normal response. Our results suggest that NMDA receptors are required for dopamine to exert its modulatory effects on the activity of the octanol avoidance circuit.

## Materials and Methods

### Strains

All strains were maintained on standard NGM agar with OP50 *E. coli* bacteria at 20 or 25°C, as described [37]. Alleles used in this study include: *cat-2 (e1112)* [38], *eat-4 (ky5)* [14], *glr-1 (n2461)* [26], *glr-1 (ky176)* [27], *nmr-1 (ak4)* [39], *glr-2 (ak10)* [40], *dop-1 (vs100)*, *dop-2 (vs105)*, *dop-3(vs106)* [41], and *dat-1 (ok157)*. Strains containing mutant alleles were obtained from the *Caenorhabditis* Genetics Center (St. Paul, MN), and strains harboring multiple mutations were generated using standard genetic crosses and PCR genotyping (see below). *glr-1 (ky176) glr-2 (ak10)* and *nmr-1; lin-15 akEx118 [gfp::nmr-1(+), lin-15(+)]* [39] were generous gifts from Villu Maricq (Univ. of Utah).

### PCR genotyping

Genotyping of all mutant alleles was performed using PCR or RFLPs. The *cat-2 (e1112)* and *glr-1 (n2461)* alleles were genotyped on the basis of *Dde*I and *Bsr*I RFLPs, respectively. All other alleles are deletion alleles and were genotyped using triplex PCR. Primer sequences, PCR conditions, and RFLP genotyping details are all available upon request.

### Behavioral assays

**Figure 1:**
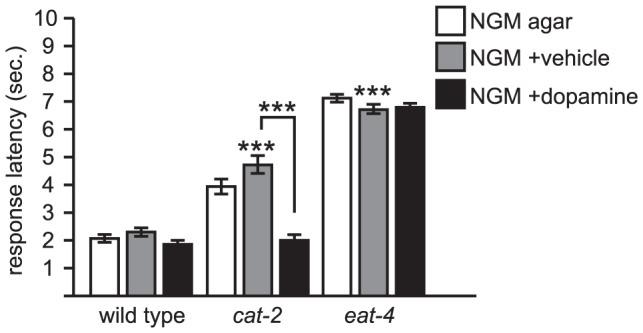
Dopamine is required for normal octanol avoidance response. *cat-2* codes for tyrosine hydroxylase, a key enzyme in the dopamine biosynthetic pathway [38]. *cat-2* mutants are defective for octanol response, but are fully restored to wild type response latencies when tested with exogenous dopamine. *eat-4* codes for a vesicular glutamate transporter that loads glutamate into synaptic vesicles [14]. *eat-4* mutants respond substantially slower than *cat-2* mutants, and are resistant to exogenous dopamine. Both *cat-2* and *eat-4* mutations are predicted nulls. In this and all the following figures, dopamine was added directly to the surface of standard NGM agar plates; distilled water was used as the vehicle control (see Materials and Methods for details). Sample sizes and detailed numbers are available in Data S1. Error bars indicate S.E.M. Comparisons are to wild type unless otherwise noted. ***p<0.0001.

The Sos ("smell-on-a-stick") assay was performed on standard nematode growth medium (NGM) plates with no food, essentially as described [9], [42]. Briefly, a paintbrush hair taped to a glass Pasteur pipette was dipped in 100% undiluted octanol (1-octanol, Sigma-Aldrich) and placed in front of a forward moving animal's nose. Response latency was scored as the time (sec.) from presentation to the initiation of a reversal. Only reversals were scored, as determined by observing backward movement of the tail. A halt of forward motion or a withdrawal of only the head was not scored. For time to spontaneous reversals, the assay was performed as described above, except an empty hair with no octanol was used. To facilitate keeping time while the experimenter was looking through a stereomicroscope during the assay, a standard metronome set to 60 bpm was used as an audible timer. Assays were halted at 20 seconds to account for spontaneous reversals [43]. We found this assay to be highly sensitive to unknown environmental variables; there was no obvious correlation with variations in temperature, humidity, or light cycle (data not shown). In addition, strong ambient odors (e.g., microwave popcorn or coffee from nearby offices) affected *C. elegans* behavior (data not shown). Therefore, we performed pilot control experiments for the N2 and *cat-2* strains at the start of each day when behavioral tests for other strains were planned. If these two control strains did not display the modulatory behavior as shown in Fig. 1, further behavioral experiments were not performed and any data generated on that day were discarded. Because of this variability, we present the N2 and *cat-2* control data for each experiment. Results are typically pooled from several experiments conducted on different days to control for environmental variability, and are presented as mean ± S.E.M.; statistical significance was measured using an unpaired Student's t-test. For brevity in the figures, statistical analysis for the wild type and *cat-2* control experiments are only shown in Fig. 1, but comparable statistical results were obtained for all experiments (see Data S1 for details).

Basal locomotion rate was visually scored by manually counting body bends of freely moving animals on NGM agar plates with no food in 10 sec. bins. The data were then normalized to body bends per minute.

### Exogenous dopamine assays

To treat animals with exogenous dopamine, 40 µL of a freshly prepared 1 M dopamine solution in distilled water was spread onto a standard NGM plate and allowed to briefly dry in a laminar flow hood. The plates were shielded from ambient light and used within 5-10 minutes. An equal volume of distilled water was used for vehicle only controls.

## Results

### Dopamine is required for normal octanol response

To determine whether dopamine plays a role in modulating octanol response, we tested dopamine-deficient *cat-2* mutant animals. *cat-2* codes for tyrosine hydroxylase, an enzyme essential for dopamine biosynthesis [38]. Based on its position within the biosynthetic pathway, loss of function in *cat-2* should in principle not affect the levels of any other catecholamines or serotonin [44]. Although the *cat-2 (e1112)* mutant allele we used is a nonsense mutation predicted to truncate the protein coding sequence prior to the active site of the enzyme and is thus likely to be a null allele, this strain still has 30–40% of wild type levels of dopamine [10], presumably due to alternate biosynthetic pathways [12]. It is unknown whether any of this residual dopamine is accessible to the nervous system.

Under our assay conditions, we found that wild type *C. elegans* respond to octanol on average within about 2 seconds, whereas *cat-2* mutant animals responded around 4 seconds (Fig. 1). The basal rate of locomotion and time to spontaneous reversal was not significantly different between the two strains with or without exogenous dopamine (Table 1). To determine the role of all glutamatergic signaling in this behavioral response, we tested *eat-4* mutant animals, which do not load glutamate into presynaptic vesicles and are therefore deficient for glutamatergic signaling [14]. *eat-4* mutant animals responded to octanol on average at around 7-8 seconds (Fig. 1), which is significantly greater than the latency to reversal of wild type and *cat-2* animals from mock octanol-less presentation (Table 1). Thus, *cat-2* animals still retain the ability to respond to octanol via glutamatergic signaling, but that response is delayed.

**Table 1:** Basal locomotion rates and time to spontaneous reversals are unaffected in dopamine and glutamate signaling mutants.

basal locomotion rate		
strain	n	body bend/min.
wild type	15	23.6±1.7
wild type (+ dopamine)	15	21.2±1.4
*cat-2*	15	20.0±1.3
*cat-2* (+ dopamine)	15	21.2±1.3
*glr-1*	40	25.5±1.6
*glr-2*	40	25.3±1.7
*nmr-1*	40	26.4±1.6

time to spontaneous reversal		
strain	n	average time to reversal (sec.)
wild type	10	18.8±1.2
*cat-2*	10	17.3±1.9
*glr-1*	10	14.7±2.7
*glr-2*	10	16.4±2.4
*nmr-1*	10	14.7±2.7

See Materials and Methods for technical details. In all cases there was no statistically significant difference between any of the strains tested and wild type. Data are presented as mean ± S.E.M. The *glr-1* allele used was *ky176*.

To determine if the behavioral deficit observed in *cat-2* mutant animals can be attributed to decreased dopamine levels, animals were tested in the presence of exogenous dopamine. Dopamine was applied to the agar plates upon which the animals crawled by dissolving it in distilled water and spreading the solution directly on the agar surface; vehicle only (i.e., distilled water alone) was used as a control. Exogenous dopamine fully restored normal response to *cat-2* animals, but had no effect on wild type or *eat-4* animals (Fig. 1). It is therefore unlikely that any behavioral deficits we observed were due to long-term developmental defects in *cat-2* mutant animals. We also tested *dat-1* mutant animals, in which loss of function in the DAT-1 dopamine reuptake transporter presumably causes an increase in endogenous dopamine levels. *dat-1* mutants had no obvious defect in octanol response (data not shown). These results suggest that dopamine modulates the octanol avoidance behavior in *C. elegans*, likely by potentiating neural activity in the octanol sensing neurons (ASH and others), premotor interneurons, or other cells that provide or receive input from these neurons.

### Dopamine modulation of octanol response does not require GLR-1 or GLR-2

Because ASH sensory neurons make glutamatergic synapses with the premotor interneurons, we tested for genetic interactions between dopamine signaling and glutamate signaling via specific postsynaptic glutamate receptors. GLR-1 and GLR-2 are AMPA/kainate-like ionotropic glutamate receptor subunits that are expressed in (among other cells) the aforementioned premotor interneurons that regulate forward and backward locomotion. Based on GFP reporter studies, GLR-1 is expressed in all five pairs of premotor interneurons, whereas GLR-2 is co-expressed with GLR-1 in all the premotor interneurons except AVB [24]. Basal locomotion rates and times to spontaneous reversal were not significantly different between wild type, *glr-1*, and *glr-2* animals (Table 1).

**Figure 2:**
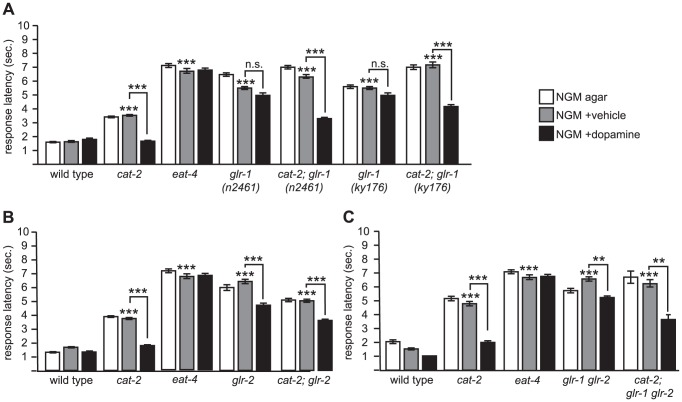
The AMPA/kainate receptors GLR-1 and GLR-2 are part of the neuronal pathway but are not required for dopamine modulation of octanol response. *glr-1* and *glr-2* code for AMPA/kainate ionotropic glutamate receptor subunits expressed in premotor interneurons of the octanol avoidance circuit. (A) *glr-1* is not required for dopamine sensitivity. *glr-1 (n2461)* is a nonsense mutation resulting in a genetic null [26], and *glr-1 (ky176)* is a Tc1 excision allele with dominant negative properties [27]. (B) *glr-2* is not required for dopamine sensitivity. *glr-2* refers to *ak10*, a deletion allele [40]. (C) *glr-1 glr-2* double mutants are resistant to exogenous dopamine. *glr-1 glr-2* is a double mutant of the *ky176* and *ak10* alleles. Also see Fig. 1 legend for technical details. **p<0.001; ***p<0.0001; n.s., not significant.

We tested two different alleles of *glr-1*. *glr-1 (ky176)* is a Tc1 transposon excision allele and is partially dominant negative in a nose touch reversal assay [27], and *glr-1 (n2461)* is a nonsense mutation that functions as a genetic null [26]. Strains carrying either mutation showed increased response latency to octanol, indicating a defect in the response (Fig. 2A). The response latency of the *glr-1* mutants was close to (or just slightly faster than) *eat-4* mutants, indicating that the deficit in octanol response in *glr-1* mutants can largely be accounted for by a loss in glutamatergic signaling. Exogenous dopamine did not improve response latency for either mutant. These results are consistent with glutamatergic signaling from ASH neurons to the premotor interneurons playing a role in the octanol avoidance response.

We then examined the response of animals carrying *glr-1* and *cat-2* mutations. *cat-2; glr-1* double mutant animals had increased response latency to octanol (Fig. 2A), similar to *glr-1* single mutants. Response latency decreased significantly in the presence of exogenous dopamine (Fig. 2A). Again, results were similar for both *glr-1* alleles. Thus, GLR-1 is required for normal octanol response, but when *glr-1* and *cat-2* loss-of-function mutations are combined, exogenous dopamine improves response latency to octanol.

We carried out a similar analysis for *glr-2*. *glr-2 (ak10)* is a deletion allele that is a predicted molecular null [40]. As with *glr-1* mutants, *glr-2* mutant animals also showed a similar increase in response latency as *glr-1* mutant animals that was comparable to *eat-4* mutants, indicating that behavioral defect in *glr-2* mutants could largely be accounted for by a deficit in glutamatergic signaling. *glr-2* mutant animals showed a slight but significant improvement when tested in the presence of exogenous dopamine (Fig. 2B). The *cat-2; glr-2* double mutant animals also had increased response latency that decreased in the presence of exogenous dopamine.

Since the GLR-1 and GLR-2 receptors are mostly co-expressed and are thought to function together in premotor interneurons yet appear to have slightly different roles in octanol response, we decided to examine *glr-1 glr-2* double mutants. We only tested one of the *glr-1 (ky176)* alleles by using a preexisting *glr-1 (ky176) glr-2 (ak10)* double mutant strain. We decided not to carry out a cross between the *n2461* and *ak10* alleles because the *glr-1* and *glr-2* genes are only about 0.5 map units apart on chromosome III. We found that *glr-1 glr-2* double mutant animals behaved similarly to that of *glr-2* single mutants; that is, *glr-1 glr-2* double mutant animals were weakly sensitive to exogenous dopamine, but exogenous dopamine significantly decreased the response latency of *cat-2; glr-1 glr-2* mutant animals (Fig. 2C).

Taken together, decreasing endogenous dopamine levels via mutation of the *cat-2* gene causes *C. elegans* to have an increased response latency to octanol, and that this does not require either the GLR-1 or the GLR-2 AMPA/kainate receptor subunits. Thus, while glutamate signaling via GLR-1 or GLR-2 is required for normal octanol response, neither receptor subunit mediates the neuromodulation by dopamine.

### Dopamine modulation of octanol response requires NMR-1

**Figure 3:**
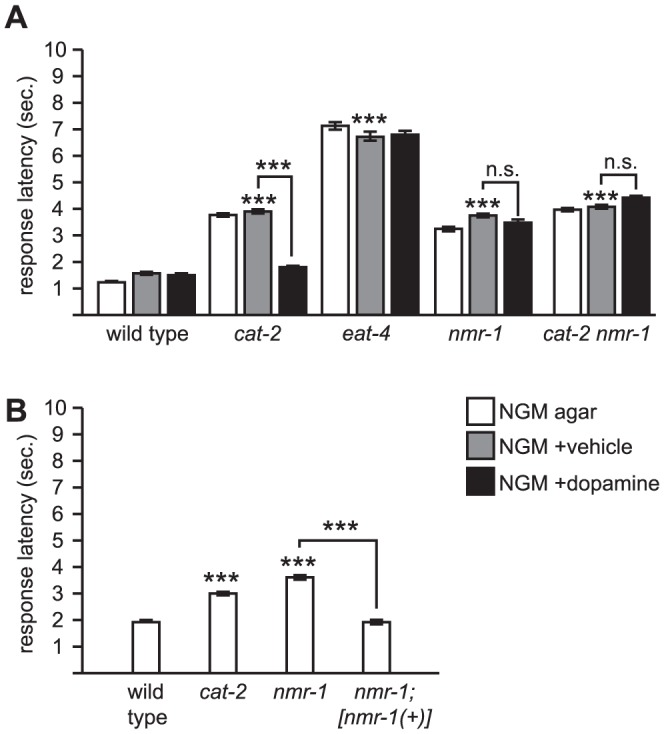
The NMDA receptor NMR-1 is required for dopamine modulation of octanol response. (A) *nmr-1* is required for dopamine sensitivity. *nmr-1* codes for a NMDA-like ionotropic glutamate receptor subunit expressed in premotor interneurons of the octanol avoidance circuit [39]. The *ak4* allele is predicted null. (B) Transgenic expression of *nmr-1(+)* restores normal octanol response to *nmr-1* mutants. *nmr-1; [nmr-1(+)]* is a previously published transgenic strain wherein *[nmr-1(+)]* refers to *akEx118*, a fully functional *gfp::nmr-1* translational fusion under the control of the endogenous *nmr-1* promoter that restores *nmr-1* function in a different behavioral paradigm [39]. Also see Fig. 1 legend for technical details. ***p<0.0001; n.s., not significant.

We then examined the role of the NMDA ionotropic glutamate receptor subunit NMR-1 by testing strains harboring the *nmr-1 (ak4)* mutation, a deletion that is a predicted molecular null [39]. The basal locomotion rate and time to spontaneous reversal of *nmr-1* animals were not significantly different than wild type animals (Table 1). *nmr-1* mutant animals had increased latency in their octanol avoidance response but the defect was not as severe as *eat-4* mutants (Fig. 3A). This defect was fully restored to wild type levels when *nmr-1* mutant animals transgenically expressed a functional GFP::NMR-1 fusion (Fig. 3B). This suggested that NMR-1 plays a lesser role (if at all) in terms of the primary signaling pathway for octanol response compared to GLR-1 and GLR-2.

We then examined whether exogenous dopamine affects the behavioral response of *nmr-1* mutants. Exogenous dopamine had no effect on the response latency of *nmr-1* mutant animals (Fig. 3A). This is in contrast to the *glr-2* mutant and the *glr-1 glr-2* double mutant, wherein exogenous dopamine decreased response latency (Fig. 2C). We then examined *cat-2 nmr-1* double mutant animals. We did not observe any additive effect on the response latency; the response latency of the double mutant was about the same as the *cat-2* or *nmr-1* single mutants. More importantly, *cat-2 nmr-1* double mutant animals were insensitive to exogenous dopamine (Fig. 3A). Thus, with regard to dopamine sensitivity, *nmr-1* is epistatic to *cat-2*, suggesting that *nmr-1* and *cat-2* are functioning in a common genetic pathway.

### *nmr-1* is epistatic to *glr-1* and *glr-2*

The genetic interaction between *cat-2* and *nmr-1* suggests that *nmr-1* may be a downstream target for dopaminergic modulation of the octanol response circuit. One prediction of this model is that *nmr-1* should be required for dopamine modulation of *C. elegans* octanol avoidance under any conditions. To test this, we generated animals that lacked both AMPA/kainate and NMDA ionotropic glutamate receptors, and determined whether or not their avoidance responses to octanol could be modulated by dopamine.

**Figure 4:**
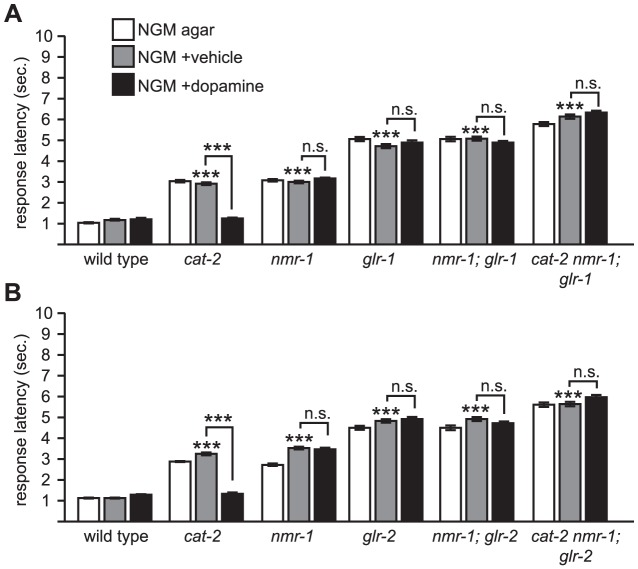
*nmr-1* is epistatic to *glr-1* and *glr-2*. (A) *glr-1* sensitivity to dopamine depends on *nmr-1*. The *glr-1* allele used for this experiment was *glr-1 (ky176)*. Compared to Fig. 2A, wherein *cat-2; glr-1* animals are sensitive to exogenous dopamine, *cat-2 nmr-1; glr-1* animals are resistant to exogenous dopamine. (B) *glr-2* sensitivity to dopamine depends on *nmr-1*. Compared to Fig. 2A, wherein *cat-2; glr-2* animals are sensitive to exogenous dopamine, *cat-2 nmr-1; glr-2* animals are resistant to exogenous dopamine. Also see Fig. 1 legend for technical details. ***p<0.0001; n.s., not significant.

*nmr-1; glr-1* mutant animals had increased response latency to octanol compared to wild type, and their response did not improve with exogenous dopamine, as expected (Fig. 4A). Similarly, *cat-2 nmr-1; glr-1* mutant animals also had increased response latencies to octanol. However, in contrast to *cat-2; glr-1* (see Fig. 2A), these triple mutant animals were insensitive to exogenous dopamine (Fig. 4A). Similar results were obtained for *glr-2* (Fig. 4B). These data indicate that *nmr-1* is required for dopamine modulation of octanol response and is consistent with a model wherein *nmr-1* is a downstream target of dopamine modulation of octanol avoidance.

### DOP-1, DOP-2, and DOP-3 are redundantly required for dopamine modulation of octanol response

**Figure 5:**
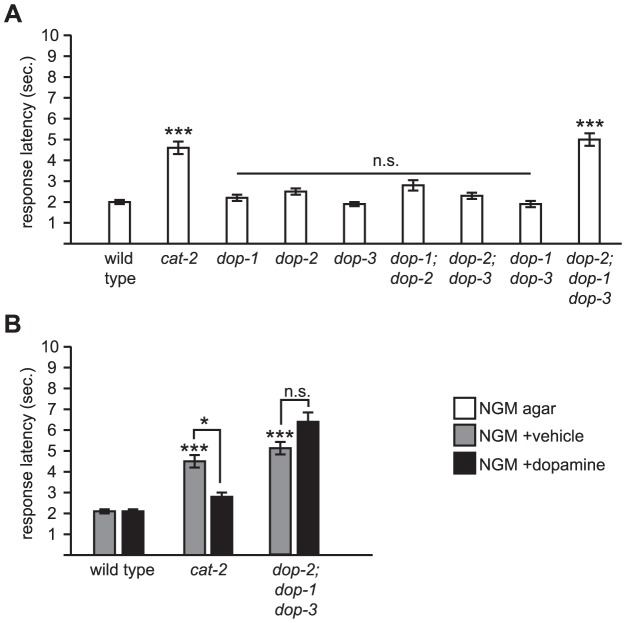
DOP-1, DOP-2, and DOP-3 are redundantly required for modulation of octanol response. (A) *dop-1*, *dop-2*, and *dop-3* code for metabotropic G-protein coupled dopamine receptors [41]. All alleles used are deletions (predicted nulls). Only the *dop-2; dop-1 dop-3* triple mutant showed a statistically significant defect for octanol response. (B) The *dop-2; dop-1 dop-3* triple mutant is resistant to exogenous dopamine. Also see Fig. 1 legend for technical details. *p<0.05, ***p<0.0001; n.s., not significant.

To determine if any known dopamine receptors are involved in modulation of octanol response, we tested deletion mutants of *dop-1*, *dop-2*, and *dop-3*, either singly or in combination. Interestingly, none of the single or double mutants displayed an obvious octanol response defect (Fig. 5A). However, the *dop-2; dop-1 dop-3* triple mutant had increased response latency, similar to the *cat-2* mutant (Fig. 5A); as expected, it was also resistant to exogenous dopamine (Fig. 5B). Thus, DOP-1, DOP-2, and DOP-3 are redundantly required for dopamine modulation of octanol response.

## Discussion

We have found that the avoidance behavior to octanol in *C. elegans* is modulated by the biogenic monoamine dopamine. In *cat-2* mutant animals wherein dopamine levels are decreased, response latency is increased; this is fully restored by assessing avoidance response in the presence of exogenous dopamine. While the AMPA/kainate ionotropic glutamate receptor subunits GLR-1 and GLR-2 are required for normal octanol response, exogenous dopamine improved response latency. Loss of function of the NMDA ionotropic glutamate receptor subunit NMR-1 also resulted in increased response latency to octanol, albeit this defect was not as severe compared to *glr-1* and *glr-2* mutants, suggesting that *nmr-1* plays a lesser role in the primary sensory pathway, if any. However, *nmr-1* and *cat-2 nmr-1* mutants were resistant to exogenous dopamine. Furthermore, when *glr-1* or *glr-2* mutations were combined with *nmr-1* in a *cat-2* mutant background, animals were also resistant to exogenous dopamine. Taken together, our results indicate that *C. elegans* response to octanol, a behavior that in part requires glutamatergic signaling via AMPA/kainate ionotropic glutamate receptors, has a separate non-glutamatergic component (likely peptidergic) the activity of which is modulated by dopamine, and that this modulation requires NMR-1.

Some prior studies have explored the role of dopamine in octanol avoidance, albeit in somewhat different behavioral paradigms. Dopamine appears to attenuate response to dilute octanol (i.e., 30% v/v in EtOH) [45]. Interestingly, observing this attenuation depends on reduced response to dilute octanol, but in our hands *C. elegans* responded robustly to dilute octanol off food. (In general we have found that *C. elegans* response to octanol is more robust than previously reported studies, and this may be due to local environmental effects that are not fully understood; data not shown). However, it is also interesting to note that for response to dilute octanol simultaneous loss of *dop-1*, *dop-2*, and *dop-3* phenocopied *cat-2* mutants [45], similar to what we report herein. This suggests that either DOP-1, DOP-2, and/or DOP-3 function redundantly to each other cell autonomously, that their expression pattern changes when one or more of the genes is mutant, or that they function redundantly but non-cell autonomously in a distributed network of neurons within the circuit. The resolution of these questions will await reagents with better resolution than GFP reporters for dopamine receptors, which currently do not report expression in *C. elegans* head neurons reliably. For instance, the dopamine receptor DOP-3, acting cell autonomously in ASH neurons, may be involved in this attenuation to dilute octanol [46]. Also, the DOP-4 dopamine receptor was shown to also play a cell-autonomous role in ASH-mediated responses to Cu^2+^, wherein dopamine potentiates Ca^2+^ currents in ASH neurons in a DOP-4 dependent fashion [8]. In both cases, GFP reporters failed to indicate dopamine receptor expression in ASH neurons despite functional evidence of that expression.

What is the site of action for dopamine in modulating octanol response? The studies referenced above suggest that the obvious location is the ASH neurons. Consistent with this, we and others have found that Ca^2+^ transients in ASH neurons are altered in *cat-2* mutants compared to wild type when animals are stimulated by high osmolarity [8](P. Turturro, C. Kunkel, and M.Y.C., in preparation). However, this does not preclude the possibility that dopamine may also be acting on other neurons. Indeed, our results showing that NMR-1 is required for dopamine modulation of octanol response suggests that the premotor interneurons may also be a site of action, although to date dopamine receptor expression has not yet been demonstrated in these neurons using GFP reporter genes. Dopamine receptors, in particular DOP-1 and DOP-3, are expressed and function in motor neurons to modulate the basal slowing response [41], but we do not think that the motor neurons are a likely candidate for a cellular site of action for modulating octanol response, because the amount of exogenous dopamine we used in this study does not affect locomotion rates (Table 1), and the concentration of dopamine used in the motor neuron study is 10-fold greater than what we used [41]. Furthermore, NMR-1 is not expressed in motor neurons, at least based on GFP reporter studies [24]. Our data indicate that no single dopamine receptor we tested is solely responsible for the modulatory activity, suggesting that dopamine modulation may be redundant among different receptor subunits or that it may be distributed among several neurons. Recently, dopamine-gated Cl^−^ channels have been reported in *C. elegans* [47] but their biological function remains unclear.

Depending on the site of action, dopamine may exert its modulatory effect on the response circuit in different ways. *nlp-3*-encoded neuropeptides released from ASH neurons promote avoidance to dilute octanol in the presence of food via a GSA-1 G_s_α dependent mechanism. This also requires the putative neuropeptide receptor NPR-17 [21]. Thus, if dopamine acts on ASH neurons it may promote increased neuropeptide release in a UNC-31/CAPS dependent mechanism [48], perhaps by signaling through DOP-3 and/or DOP-4. A peptidergic component to the primary synaptic signaling between ASH and the premotor interneurons is likely, given that *eat-4* mutants still have a significant residual response to octanol. However, our experiments with undiluted octanol were performed off food, which may not involve GSA-1 but rather EGL-30/G_q_α, which is the case for response to dilute octanol [21]. Alternatively, dopamine may act on premotor interneurons by activating NMR-1. This may simply increase postsynaptic sensitivity to NMDA signaling and/or result in additional intracellular Ca^2+^-dependent events via NMR-1 mediated conductances, perhaps through direct phosphorylation of NMDA receptor subunits via cAMP/PKA dependent mechanisms as seen in mammalian neurons [49]. Or, dopamine receptors may physically interact with NMDA receptors [35]. Finally, ASH may act in novel ways on other neurons that are neither sensory nor premotor. The aforementioned neuropeptide receptor NPR-17 does not appear to be expressed in premotor interneurons but rather in the AVG interneuron and several tail neurons [21], and interestingly NMR-1 is also expressed in AVG neurons [24]. AVG neurons have a role as a pioneering neuron for the ventral cord axon tract during development [50], but they have few synaptic connections and little is known about their function in adult animals. Finally, given the redundancy of dopamine receptors we and others [45] have observed, it is possible that some or all of these mechanisms are used to modulate *C. elegans* behaviors. The emerging picture is that the modulation of even a relatively simple behavior such as the avoidance response involves a complex, multicellular pathway involving multiple neurotransmitter systems, of which *nmr-1* is just one component.

## Supporting Information

Data S1 **Excel spreadsheet containing raw data for all behavioral assays.** Mean response latency, S.E.M., sample size, and p values (where applicable) are given.
(XLSX)

## References

[1] Jones S, Bonci A (2005) Synaptic plasticity and drug addiction. Curr Opin Pharmacol 5: 20–25 10.1016/j.coph.2004.08.011 15661621

[2] Zhuo M (2000) Silent glutamatergic synapses and long-term facilitation in spinal dorsal horn neurons. Prog Brain Res 129: 101–113 10.1016/S0079-6123(00)29008-0 11098684

[3] Kindt KS, Quast KB, Giles AC, De S, Hendrey D, et al (2007) Dopamine mediates context-dependent modulation of sensory plasticity in *C. elegans* . Neuron 55: 662–676 10.1016/j.neuron.2007.07.023 17698017

[4] Kang L, Gao J, Schafer WR, Xie Z, Xu XZS (2010) *C. elegans* TRP family protein TRP-4 is a pore-forming subunit of a native mechanotransduction channel. Neuron 67: 381–391 10.1016/j.neuron.2010.06.032 20696377 PMC2928144

[5] Sawin ER, Ranganathan R, Horvitz HR (2000) *C. elegans* locomotory rate is modulated by the environment through a dopaminergic pathway and by experience through a serotonergic pathway. Neuron 26: 619–631. 10896158 10.1016/s0896-6273(00)81199-x

[6] Loer CM, Kenyon CJ (1993) Serotonin-deficient mutants and male mating behavior in the nematode *Caenorhabditis elegans* . J Neurosci 13: 5407–5417. 8254383 10.1523/JNEUROSCI.13-12-05407.1993 PMC6576401

[7] Hills T (2004) Dopamine and glutamate control area-restricted search behavior in *Caenorhabditis elegans* . J Neurosci 24: 1217–1225 10.1523/JNEUROSCI.1569-03.2004 14762140 PMC6793574

[8] Ezcurra M, Tanizawa Y, Swoboda P, Schafer WR (2011) Food sensitizes *C. elegans* avoidance behaviours through acute dopamine signalling. EMBO J 30: 1110–1122. 21304491 10.1038/emboj.2011.22 PMC3061029

[9] Troemel ER, Chou JH, Dwyer ND, Colbert HA, Bargmann CI (1995) Divergent seven transmembrane receptors are candidate chemosensory receptors in *C. elegans* . Cell 83: 207–218. 7585938 10.1016/0092-8674(95)90162-0

[10] Sanyal S, Wintle RF, Kindt KS, Nuttley WM, Arvan R, et al (2004) Dopamine modulates the plasticity of mechanosensory responses in *Caenorhabditis elegans* . EMBO J 23: 473–482 10.1038/sj.emboj.7600057 14739932 PMC1271763

[11] Hilliard MA, Bergamasco C, Arbucci S, Plasterk RHA, Bazzicalupo P (2004) Worms taste bitter: ASH neurons, QUI-1, GPA-3 and ODR-3 mediate quinine avoidance in *Caenorhabditis elegans* . EMBO J 23: 1101–1111 10.1038/sj.emboj.7600107 14988722 PMC380969

[12] Rios M, Habecker B, Sasaoka T, Eisenhofer G, Tian H, et al (1999) Catecholamine synthesis is mediated by tyrosinase in the absence of tyrosine hydroxylase. J Neurosci 19: 3519–3526. 10212311 10.1523/JNEUROSCI.19-09-03519.1999 PMC6782225

[13] Sambongi Y, Nagae T, Liu Y, Yoshimizu T, Takeda K, et al (1999) Sensing of cadmium and copper ions by externally exposed ADL, ASE, and ASH neurons elicits avoidance response in *Caenorhabditis elegans* . Neuroreport 10: 753–757. 10208543 10.1097/00001756-199903170-00017

[14] Lee RY, Sawin ER, Chalfie M, Horvitz HR, Avery L (1999) EAT-4, a homolog of a mammalian sodium-dependent inorganic phosphate cotransporter, is necessary for glutamatergic neurotransmission in *Caenorhabditis elegans* . J Neurosci 19: 159–167. 9870947 10.1523/JNEUROSCI.19-01-00159.1999 PMC3759158

[15] Culotti JG, Russell RL (1978) Osmotic avoidance defective mutants of the nematode *Caenorhabditis elegans* . Genetics 90: 243–256. 730048 10.1093/genetics/90.2.243 PMC1213887

[16] Hart AC, Kass J, Shapiro JE, Kaplan JM (1999) Distinct signaling pathways mediate touch and osmosensory responses in a polymodal sensory neuron. J Neurosci 19: 1952–1958. 10066248 10.1523/JNEUROSCI.19-06-01952.1999 PMC6782580

[17] Kaplan JM, Horvitz HR (1993) A dual mechanosensory and chemosensory neuron in *Caenorhabditis elegans* . Proc Natl Acad Sci USA 90: 2227–2231. 8460126 10.1073/pnas.90.6.2227 PMC46059

[18] Chalfie M, Sulston JE, White JG, Southgate E, Thomson JN, et al (1985) The neural circuit for touch sensitivity in *Caenorhabditis elegans* . J Neurosci 5: 956–964. 3981252 10.1523/JNEUROSCI.05-04-00956.1985 PMC6565008

[19] Wittenburg N, Baumeister R (1999) Thermal avoidance in *Caenorhabditis elegans*: an approach to the study of nociception. Proc Natl Acad Sci USA 96: 10477–10482. 10468634 10.1073/pnas.96.18.10477 PMC17914

[20] Chao MY, Komatsu H, Fukuto HS, Dionne HM, Hart AC (2004) Feeding status and serotonin rapidly and reversibly modulate a *Caenorhabditis elegans* chemosensory circuit. Proc Natl Acad Sci USA 101: 15512–15517 10.1073/pnas.0403369101 15492222 PMC524441

[21] Harris G, Mills H, Wragg R, Hapiak V, Castelletto M, et al (2010) The monoaminergic modulation of sensory-mediated aversive responses in *Caenorhabditis elegans* requires glutamatergic/peptidergic cotransmission. J Neurosci 30: 7889–7899 10.1523/JNEUROSCI.0497-10.2010 20534837 PMC3005568

[22] Mills H, Wragg R, Hapiak V, Castelletto M, Zahratka J, et al (2012) Monoamines and neuropeptides interact to inhibit aversive behaviour in *Caenorhabditis elegans* . EMBO J 31: 667–678 10.1038/emboj.2011.422 22124329 PMC3273394

[23] Hapiak V, Summers P, Ortega A, Law WJ, Stein A, et al (2013) Neuropeptides amplify and focus the monoaminergic inhibition of nociception in *Caenorhabditis elegans* . J Neurosci 33: 14107–14116 10.1523/JNEUROSCI.1324-13.2013 23986246 PMC3756756

[24] Brockie PJ, Madsen DM, Zheng Y, Mellem J, Maricq AV (2001) Differential expression of glutamate receptor subunits in the nervous system of *Caenorhabditis elegans* and their regulation by the homeodomain protein UNC-42. J Neurosci 21: 1510–1522. 11222641 10.1523/JNEUROSCI.21-05-01510.2001 PMC6762961

[25] Kano T, Brockie PJ, Sassa T, Fujimoto H, Kawahara Y, et al (2008) Memory in *Caenorhabditis elegans* is mediated by NMDA-type ionotropic glutamate receptors. Curr Biol 18: 1010–1015 10.1016/j.cub.2008.05.051 18583134 PMC2645413

[26] Hart AC, Sims S, Kaplan JM (1995) Synaptic code for sensory modalities revealed by *C. elegans* GLR-1 glutamate receptor. Nature 378: 82–85 10.1038/378082a0 7477294

[27] Maricq AV, Peckol E, Driscoll M, Bargmann CI (1995) Mechanosensory signalling in *C. elegans* mediated by the GLR-1 glutamate receptor. Nature 378: 78–81 10.1038/378078a0 7477293

[28] Nathoo AN, Moeller RA, Westlund BA, Hart AC (2001) Identification of neuropeptide-like protein gene families in *Caenorhabditis elegans* and other species. Proc Natl Acad Sci USA 98: 14000–14005 10.1073/pnas.241231298 11717458 PMC61156

[29] Cepeda C, André VM, Jocoy EL, Levine MS (2009) NMDA and Dopamine: Diverse Mechanisms Applied to Interacting Receptor Systems. In: VanDongen AM, editor. Biology of the NMDA Receptor. Boca Raton (FL): CRC Press. pp. 41–58. 21204414

[30] Blank T, Nijholt I, Teichert U, Kügler H, Behrsing H, et al (1997) The phosphoprotein DARPP-32 mediates cAMP-dependent potentiation of striatal N-methyl-D-aspartate responses. Proc Natl Acad Sci USA 94: 14859–14864. 9405704 10.1073/pnas.94.26.14859 PMC25128

[31] Chergui K, Lacey MG (1999) Modulation by dopamine D1-like receptors of synaptic transmission and NMDA receptors in rat nucleus accumbens is attenuated by the protein kinase C inhibitor Ro 32-0432. Neuropharmacology 38: 223–231. 10218863 10.1016/s0028-3908(98)00187-7

[32] Chen G, Greengard P, Yan Z (2004) Potentiation of NMDA receptor currents by dopamine D1 receptors in prefrontal cortex. Proc Natl Acad Sci USA 101: 2596–2600. 14983054 10.1073/pnas.0308618100 PMC356995

[33] Cepeda C, Buchwald NA, Levine MS (1993) Neuromodulatory actions of dopamine in the neostriatum are dependent upon the excitatory amino acid receptor subtypes activated. Proc Natl Acad Sci USA 90: 9576–9580. 7692449 10.1073/pnas.90.20.9576 PMC47612

[34] Hernandez-Lopez S, Tkatch T, Perez-Garci E, Galarraga E, Bargas J, et al (2000) D2 dopamine receptors in striatal medium spiny neurons reduce L-type Ca2+ currents and excitability via a novel PLCβ1-IP3-calcineurin-signaling cascade. Journal of Neuroscience 20: 8987–8995. 11124974 10.1523/JNEUROSCI.20-24-08987.2000 PMC6773013

[35] Lee FJS, Xue S, Pei L, Vukusic B, Chéry N, et al (2002) Dual regulation of NMDA receptor functions by direct protein-protein interactions with the dopamine D1 receptor. Cell 111: 219–230. 12408866 10.1016/s0092-8674(02)00962-5

[36] Fiorentini C, Gardoni F, Spano P, Di Luca M, Missale C (2003) Regulation of dopamine D1 receptor trafficking and desensitization by oligomerization with glutamate N-methyl-D-aspartate receptors. J Biol Chem 278: 20196–20202 10.1074/jbc.M213140200 12646556

[37] Stiernagle T (2006) Maintenance of *C. elegans*. WormBook. doi:10.1895/wormbook.1.101.1. 10.1895/wormbook.1.101.1 PMC4781397 18050451

[38] Lints R, Emmons SW (1999) Patterning of dopaminergic neurotransmitter identity among *Caenorhabditis elegans* ray sensory neurons by a TGFβ family signaling pathway and a Hox gene. Development 126: 5819–5831. 10572056 10.1242/dev.126.24.5819

[39] Brockie PJ, Mellem JE, Hills T, Madsen DM, Maricq AV (2001) The *C. elegans* glutamate receptor subunit NMR-1 is required for slow NMDA-activated currents that regulate reversal frequency during locomotion. Neuron 31: 617–630. 11545720 10.1016/s0896-6273(01)00394-4

[40] Mellem JE, Brockie PJ, Zheng Y, Madsen DM, Maricq AV (2002) Decoding of polymodal sensory stimuli by postsynaptic glutamate receptors in *C. elegans* . Neuron 36: 933–944. 12467596 10.1016/s0896-6273(02)01088-7

[41] Chase DL, Pepper JS, Koelle MR (2004) Mechanism of extrasynaptic dopamine signaling in *Caenorhabditis elegans* . Nat Neurosci 7: 1096–1103 10.1038/nn1316 15378064

[42] Hart A (2006) Behavior. WormBook: 1–67. doi:10.1895/wormbook.1.87.1.

[43] Chao MY, Larkins-Ford J, Tucey TM, Hart AC (2005) lin-12 Notch functions in the adult nervous system of *C. elegans* . BMC Neurosci 6: 45 10.1186/1471-2202-6-45 16011804 PMC1181819

[44] Chase DL, Koelle MR (2007) Biogenic amine neurotransmitters in *C. elegans*. WormBook : 1–15. doi:10.1895/wormbook.1.132.1. 10.1895/wormbook.1.132.1 PMC4781333 18050501

[45] Wragg RT, Hapiak V, Miller SB, Harris GP, Gray J, et al (2007) Tyramine and octopamine independently inhibit serotonin-stimulated aversive behaviors in *Caenorhabditis elegans* through two novel amine receptors. J Neurosci 27: 13402–13412 10.1523/JNEUROSCI.3495-07.2007 18057198 PMC6673087

[46] Ezak MJ, Ferkey DM (2010) The *C. elegans* D2-like dopamine receptor DOP-3 decreases behavioral sensitivity to the olfactory stimulus 1-octanol. PLoS ONE 5: e9487 10.1371/journal.pone.0009487 20209143 PMC2830454

[47] Ringstad N, Abe N, Horvitz HR (2009) Ligand-gated chloride channels are receptors for biogenic amines in *C. elegans* . Science 325: 96–100 10.1126/science.1169243 19574391 PMC2963310

[48] Speese S, Petrie M, Schuske K, Ailion M, Ann K, et al (2007) UNC-31 (CAPS) is required for dense-core vesicle but not synaptic vesicle exocytosis in *Caenorhabditis elegans* . J Neurosci 27: 6150–6162 10.1523/JNEUROSCI.1466-07.2007 17553987 PMC6672138

[49] Murphy JA, Stein IS, Lau CG, Peixoto RT, Aman TK, et al (2014) Phosphorylation of Ser1166 on GluN2B by PKA is critical to synaptic NMDA receptor function and Ca^2+^ signaling in spines. J Neurosci34: 869–879 10.1523/JNEUROSCI.4538-13.2014 PMC3891964 24431445

[50] Hutter H (2003) Extracellular cues and pioneers act together to guide axons in the ventral cord of *C. elegans* . Development 130: 5307–5318 10.1242/dev.00727 13129845

